# Chitosan as a Promising Support of a CDH Activity Preservation System for Biomedical and Industrial Applications

**DOI:** 10.3390/ijms24054535

**Published:** 2023-02-25

**Authors:** Justyna Sulej, Monika Osińska-Jaroszuk, Magdalena Jaszek, Anna Olszewska, Anna Belcarz, Wiktoria Piątek-Gołda

**Affiliations:** 1 Department of Biochemistry and Biotechnology, Institute of Biological Sciences, Maria Curie-Sklodowska University, Akademicka 19, 20-033 Lublin, Poland; 2 Department of Human Physiology, Medical University of Lublin, 11 Radziwiłowska Street, 20-080 Lublin, Poland; 3 Chair and Department of Biochemistry and Biotechnology, Medical University of Lublin, 1 Chodźki Street, 20-093 Lublin, Poland

**Keywords:** cellobiose dehydrogenase, immobilization, chitosan, antioxidant, antimicrobial, cytotoxic

## Abstract

Cellobiose dehydrogenase (CDH) is an extracellular hemoflavoprotein catalyzing the oxidation reaction of β-1,4-glycosidic-bonded sugars (lactose or cellobiose), which results in the formation of aldobionic acids and hydrogen peroxide as a byproduct. The biotechnological application of CDH requires the immobilization of the enzyme on a suitable support. As a carrier of natural origin used for CDH immobilization, chitosan seems to increase the catalytic potential of the enzyme, especially for applications as packaging in the food industry and as a dressing material in medical applications. The present study aimed to immobilize the enzyme on chitosan beads and determine the physicochemical and biological properties of immobilized CDHs obtained from different fungal sources. The chitosan beads with immobilized CDHs were characterized in terms of their FTIR spectra or SEM microstructure. The most effective method of immobilization in the proposed modification was the covalent bonding of enzyme molecules using glutaraldehyde, resulting in efficiencies ranging from 28 to 99%. Very promising results, compared to free CDH, were obtained in the case of antioxidant, antimicrobial, and cytotoxic properties. Summarizing the obtained data, chitosan seems to be a valuable material for the development of innovative and effective immobilization systems for biomedical applications or food packaging, preserving the unique properties of CDH.

## 1. Introduction

Fungi are a well-characterized group of producers of many biotechnologically important intracellular and extracellular enzymes. The best-known fungal enzymes include laccase, chitinase, esterase, manganese peroxidases, glucose oxidase, and cellobiose dehydrogenase [1,2,3,4,5,6,7]. These enzymes are applied in a wide range of industries, such as detergents, textiles, leather processing, forest products, baking, brewing, fermented products, pharmaceuticals, animal feed, fuel ethanol, and food [8,9]. In addition, fungal enzymes synthesize economically valuable compounds [10], the production of which with the use of metallic catalysts, although usually efficient, generates more byproducts and can be toxic to the environment. In contrast, the enzymes are non-toxic, biocompatible, and biodegradable, which eliminates pollution concerns [1]. In spite of many advantages, free fungal enzymes have some limitations, especially in difficult environmental conditions, such as strong acidity and/or high temperature, in which they can undergo denaturation decreasing their activity and affinity for substrates [11]. In order to improve the catalytic properties and stability of enzymes, the immobilization technique is used to optimize enzyme–substrate interactions and minimize non-specific interactions [12]. The use of immobilized enzymes in biotechnological processes allows multiple and repeated uses of a single batch of enzymes that are usually more stable than mobile enzymes. The catalyzed reaction can be controlled rapidly by removing the enzyme from the reaction solution, and contamination can be avoided by the easy separation of the enzyme from the product. The use of immobilized enzymes allows the development of multi-enzyme reaction systems, which are increasingly being used in industrial processes [13]. Various natural and synthetic matrices are used for immobilization adapted to the enzyme application. Organic and inorganic carriers such as alginate beads, glass and silica beads, activated carbon, and chitosan microsphere are most commonly used [14].

Chitosan, the product of chitin deacetylation, has gained a lot of interest as an attractive and cost-effective biomaterial for the immobilization of many enzymes used in a variety of fields, including medicine [15,16], wastewater treatment [17], agriculture [18], and biotechnology [19,20]. This polyaminosaccharide composed of β-(1–4)-linked *D*-glucosamine and *N*-acetyl-*D*-glucosamine units has several attractive properties, such as its natural origin, antibacterial activity, non-toxicity, easy modification, biodegradability, and low cost [21,22]. The presence of primary amino and hydroxyl groups in chitosan enhances the interaction between the biopolymer and enzymes, which allows the use of simple immobilization techniques, e.g., adsorption and covalent bonding [23]. The covalent bonding of the enzyme to the carrier is usually stronger and minimizes its leaching in the aqueous medium; it may also improve the biocatalyst stability and modify its catalytic properties [24].

Cellobiose dehydrogenase (CDH; EC1.1.99.18; CAZy: AA3.1) is an extracellular oxidoreductase (hemoflavoenzyme) secreted by fungi to assist lignocellulolytic enzymes in biomass degradation. CDH is a hemoflavoenzyme composed of a catalytic flavodehydrogenase (DH) domain linked to an *N*-terminal electron-transferring cytochrome (CYT) domain [25]. This enzyme is capable of catalyzing the oxidation of di- and oligosaccharides linked by β-1− 4-glycosidic bonds, including cellobiose and lactose, to the corresponding aldono-1,5-lactones, which spontaneously undergo hydrolysis to aldonic acids in an aqueous solution [26,27]. The cellobiose dehydrogenase (CDH) enzyme has been studied for many years for applications in biosensors and as an anode in biofuel cells due to its unique bioelectrochemical properties. Additionally, in the last decade, the antibiofilm, antimicrobial, and antioxidant properties of CDH have been associated with the presence of products of the CDH-catalyzed reaction consisting in the oxidation of lactose into lactobionic acid and such byproducts as hydrogen peroxide (H_2_O_2_), which are used in biomedicine as well as food and packaging industries [28]. The use of chitosan as a support for the immobilization of cellobiose dehydrogenases obtained by our research team is very reasonable given the application potential of these enzymes in both the medicine and food industries. Moreover, a very important issue concerning the application of immobilized enzymes, especially in these areas, is the potential cytotoxic effect. The use of a cross-linking agent, e.g., glutaraldehyde, can have a negative effect on healthy cells even if the matrix itself is non-toxic. This may affect the applicability of the immobilized enzyme system in a particular field.

Despite the huge potential of CDH, the research of the immobilized systems of this biocatalyst is a scientific novelty that is rare among research teams who work with this enzyme. Research on the use of cellobiose dehydrogenase immobilized on biocompatible matrices, such as chitosan, will allow expanding the application range of this enzyme. In the context of immobilization on the chitosan matrix, enzymes from *C. unicolor*, *P. sanguineus*, and *P. lindtnerii* have not been studied yet.

The aim of the present research was to apply chitosan beads as a support for the immobilization of four different fungal cellobiose dehydrogenases with varied application potential. By applying adsorption and the covalent immobilization of the enzymes and an enzyme/substrate system, the effectiveness of the immobilization process was determined. The chitosan beads with immobilized CDHs were characterized in terms of their FTIR spectra, SEM microstructure, and antioxidant, antimicrobial, and cytotoxic properties.

## 2. Results and Discussion

### 2.1. Immobilization of Cellobiose Dehydrogenase on Chitosan Beads

The use of immobilized enzymes instead of the free form of the biocatalyst has long been used for industrial purposes and offers additional advantages such as thermal stability and reusability. The immobilization of a protein with high application potential may increase the possibilities of its use in various industries, including those where it has not been used before.

**Figure 1:**
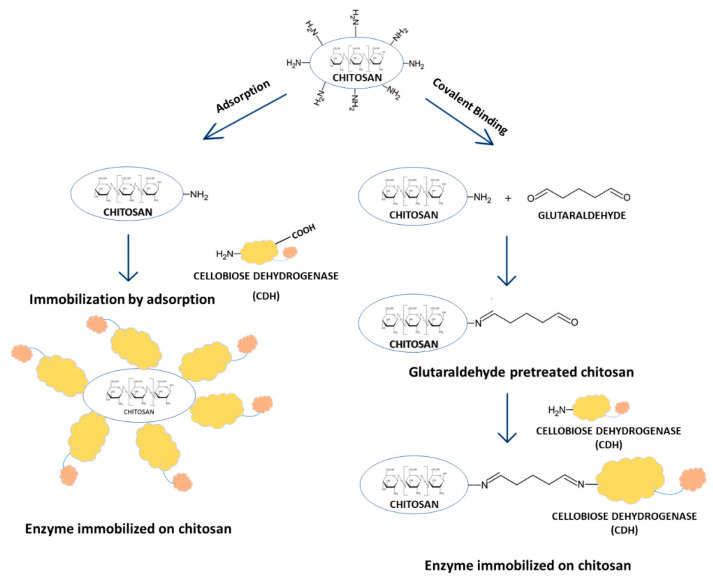
Immobilization of cellobiose dehydrogenase on chitosan through direct adsorption and covalent attachment using glutaraldehyde.

There are two main methods of enzyme immobilization: physical and chemical. In the physical method, the enzyme interacts with the carrier (support) material without forming any formal chemical bonds. The protein can be adsorbed on the surface of the support material or entrapped within. The adsorption of the enzyme on the carrier is relatively simple, but the interaction between the carrier and the protein is minimal, and these materials are susceptible to leaching [29]. In the chemical method, the enzyme binds to the carrier through different covalent or non-covalent chemical interactions [30]. The most commonly used technique for enzyme immobilization is covalent immobilization using glutaraldehyde [31]. The reaction of glutaraldehyde with chitosan allows the activation of the support with aldehyde groups that may react with the enzyme through its amino groups (ε-amino group of lysine residues and terminal amino group) and sometimes with other surface functional groups (thiols, phenols, and imidazoles) [32]. Figure 1 shows the CDH immobilization scheme.

Natural biodegradable polymers such as alginate, carrageenan, or chitosan used as matrices for immobilization have found their special application in the engineering industry or medicine due to their ability to form hydrogels, fibers, films, or beads. In this study, we investigated the immobilization of cellobiose dehydrogenase on chitosan beads and compared the efficiency of the physical and chemical immobilization of CDH on chitosan. This polymer was used by Tegl et al. in their research on the biomedical application of CDH as an antibacterial agent [33].

#### 2.1.1. Direct Adsorption

In the simplest immobilization technique, i.e., adsorption, the enzyme is bound to the carrier through hydrogen bonding, van der Waals forces, or hydrophobic interactions (physical adsorption) or through salt linkages (ionic binding) [34].

The chitosan carrier for CDH immobilization was prepared by precipitation in NaOH to form beads. The chitosan beads were immobilized by adsorption in the CDH-alone system and in the chitosan/CDH/substrate complex. Table 1 shows the differences in the protein binding efficiency and the CDH activity yield obtained from different fungi with or without the substrate. The highest activity yield of about 78% was obtained for CDH from the *C. unicolor*/lactose complex with protein binding of about 54%. Similarly, 60% efficiency of CDH immobilization on chitosan particles with the adsorption method was determined by Tegl et al. [33]. In our previous studies, we obtained higher CDH binding efficiency (from 60 to 100% depending on the enzyme tested) using the method of CDH entrapment in alginate beads [28].

#### 2.1.2. Covalent Immobilization Using Glutaraldehyde

The covalent immobilization of enzymes is one of the most widely used methods of attaching proteins to solid supports. This is associated with the stable nature of the bond preventing the enzyme from being released back into the solution [35]. However, such a strong attachment is a significant modification of the protein and can sometimes lead to changes in its hydrophilic/hydrophobic properties, which may significantly affect the efficiency of the immobilization process [29]. In our research, we used glutaraldehyde as a well-known cross-linking agent responsible for stiffening the enzyme structure and increasing its binding to the carrier [32]. A side effect of this procedure may be a remarkable decrease in the catalytic activity of the enzyme after immobilization. Nevertheless, from the practical point of view, it is much more important that biocatalyst preparations additionally crosslinked with glutaraldehyde exhibit significantly increased stability in processing conditions [36]. CDH was immobilized on chitosan beads activated with glutaraldehyde, which provided an excellent biocompatible surface since immobilization efficiency in the range of 28 to 99% for the different sources of the enzyme and enzyme/substrate complexes was achieved. Table 2 shows the immobilization yield of different variants of the immobilized enzyme. Compared to adsorption, the use of chitosan beads activated with the crosslinking agent significantly increased the efficiency of the immobilization process. Glutaraldehyde used as a crosslinking agent for the immobilization of enzymes on chitosan beads prevents the direct contact of the enzyme with the surrounding medium and enables the reagents to reach the catalytic site [37]. This technique has been used in many research studies to effectively immobilize such enzymes as lipase [38], β-galactosidase [37], or laccase [39] on chitosan beads The efficiency of the process of immobilization of various enzymes was high with values in the range of 84.7% [39] and 88.4 % [40] for laccase to 99.1% for lipase [41]. CDH immobilization has also been carried out on glutaraldehyde-modified magnetic chitosan spheres and chitosan particles used as carriers with activity yields of 61.54% [7] and 40% [42], respectively

### 2.2. Characterization of the Chitosan Beads

#### 2.2.1. FTIR

**Figure 2:**
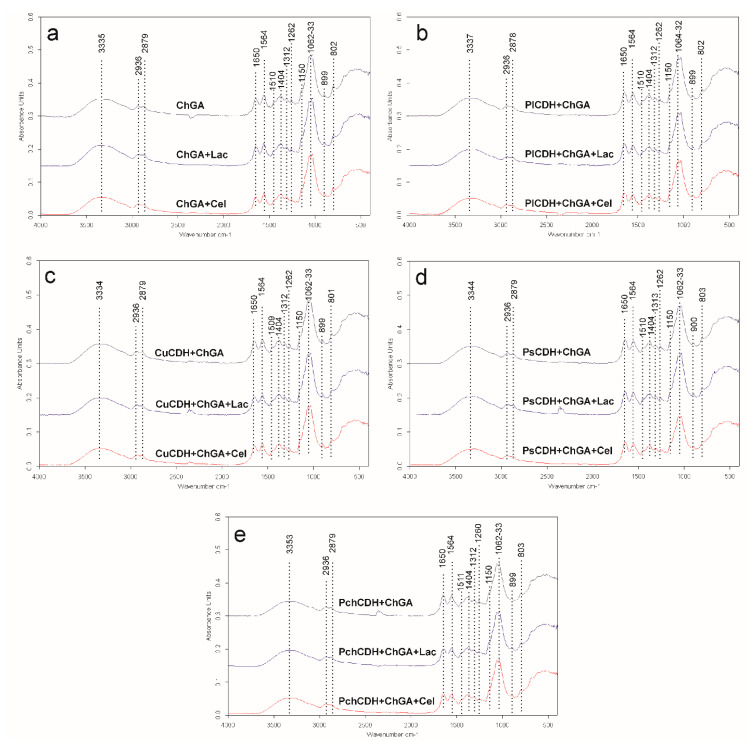
(**a**) FTIR spectra of chitosan with glutaraldehyde (ChGA) (C1-control), ChGA + Lac (C2-control), ChGA + Cel, (C3-control); (**b**) FTIR spectra chitosan with glutaraldehyde (ChGA) containing CDHs isolated from *P. lindtneri*: *Pl*CDH + ChGA, *Pl*CDH + ChGA + Lac, *Pl*CDH + ChGA + Cel; (**c**) FTIR spectra chitosan with glutaraldehyde (ChGA) containing CDHs isolated from *C. unicolor*: *Cu*CDH + ChGA, *Cu*CDH+ ChGA + Lac, *Cu*CDH + ChGA + Cel; (**d**) FTIR spectra chitosan with glutaraldehyde (ChGA) containing CDHs isolated from *P. sanguineus*: *Ps*CDH + ChGA, *Ps*CDH + ChGA + Lac, *Ps*CDH +ChGA + Cel; (**e**) FTIR spectra chitosan with glutaraldehyde (ChGA) containing CDHs isolated from *P. chrysosporium*: *Pch*CDH + ChGA, *Pch*CDH+ ChGA + Lac, *Pch*CDH + ChGA + Cel.

The spectra of all the tested samples showed significant similarities (Figure 2). A broad band of overlapping peaks of O-H and N-H stretching was recorded at 3335–3353 cm^−1^ in all spectra, which is in agreement with observations of glutaraldehyde-crosslinked chitosan reported by another research team [43]. Two bands of asymmetric and symmetric -CH_3_ and -CH_2_ vibrations were found at 2936 and 2879 cm^−1^, respectively. Li et al. (2013) found that the band of -CH_3_ vibrations shifted from 2925 cm^−1^ in pure chitosan to 2937 cm^−1^ in glutaraldehyde-crosslinked chitosan, which may suggest successive crosslinking of the chitosan polymer in the case of this study. This may be supported by the observation of a small peak at 1404 cm^−1^ in all the spectra, indicating the presence of C-N stretching vibrations. Li et al. (2013) also observed the appearance of such a peak in glutaraldehyde-crosslinked chitosan in comparison with a peak at 1438 cm^−1^ for pure chitosan. These bands may indicate successful chitosan crosslinking by glutaraldehyde. On the other hand, a very small peak was observed at 1150 cm^−1^ in the spectra of all the samples [44]. As reported by Islam et al. (2019), this band indicated the presence of asymmetric stretching vibrations of the C-O-C bridge in the glycosidic bond in pure chitosan. Islam observed that this peak was shifted to 1110 cm^−1^ in the spectra of glutaraldehyde-crosslinked chitosan. Therefore, the presence of the minimal peak of 1150 cm^−1^ in these samples may suggest the presence of traces of uncrosslinked chitosan in all the tested samples [43].

Two dominating sharp peaks at 1062 and 1033 cm^−1^ represent the O-C-O ring of chitosan [43,45]. The 1510 and 899 cm^−1^ bands may indicate the presence of N-H bending vibrations and C-C stretching vibrations (in the glucose ring), respectively [45]. The 1374 cm^−1^ band may indicate the presence of either CH_3_ bending vibrations [44] or in-plane OH bending vibrations [38]. The 1262 cm^−1^ band can be assigned to the bending vibrations of hydroxyls present in chitosan [46] or to CH_2_ wagging vibrations [47]. The 1312 cm^−1^ and 1201 cm^−1^ bands may indicate the presence of an amide III band (complex vibrations of NHCO group) [48] and OH plane deformation modes [49] observed in prawn-derived chitin.

The presence of 802 cm^−1^ bands in all the spectra is probably related to the presence of N-H wagging vibrations. Islam et al. (2019) and Vijayalakshmi et al. (2016) also observed this peak in glutaraldehyde-crosslinked chitosan but at a slightly different position (at 822 cm^−1^). This peak shows slightly higher intensity in the spectrum of glutaraldehyde-crosslinked chitosan without any additives (sample ChGA) than in the spectra of the other samples. This may be caused by the increasing percentage of substances other than the crosslinked chitosan (namely sugars and proteins) in the spectra of these samples [43,45]. In all the tested samples, two significant peaks of 1650 and 1564 cm^−1^ were observed at positions recognized as amine I and amide II bands, respectively. The amide I band is assigned to C=O stretching vibrations of the peptide bond (about 80%) with a minor contribution from C-N stretching vibration (about 20%), whereas the amide II band derives mainly from in-plane NH bending and C-N stretching vibrations (40–60% and 18–40% of the potential energy, respectively) [50]. In samples ChGA (C1-control), ChGA + Lac (C2-control), and ChGA + Cel (C3-control) (without the enzyme addition), the amide II band dominates over the amide I band. However, when the spectrum of sample ChGA was compared with the spectra of samples *Pl*CDH + ChGA, *Cu*CDH + ChGA, *Ps*CDH + ChGA, or *Pch*CDH + ChGA (with the addition of protein), the height of the amide I band increased in the latter spectra. This suggested an increase in the C=O stretching vibration content in the overall signal intensity caused by the increase in the amount of peptide bonds in the tested samples. A similar relationship was observed for the spectrum of sample ChGA + Lac versus the spectra of samples *Pl*CDH + ChGA + Lac, *Cu*CDH + ChGA + Lac, *Ps*CDH + ChGA + Lac, and *Pch*CDH + ChGA + Lac (enriched with lactose) and for the spectrum of sample ChGA + Cel versus the spectra of samples *Pl*CDH + ChGA + Cel, *Cu*CDH + ChGA + Cel, *Ps*CDH + ChGA + Cel, and *Pch*CDH + ChGA + Cel (enriched with cellobiose). The ratio of the amide I to amide II band intensity ranged from 0.81 to 0.9 for chitosan without enzymes (ChGA, ChGA + Lac, and ChGA + Cel) but increased when the proteins were added (1.07–1.33 for chitosan containing *Cu*CDH and *Pch*CDH and 1.42–1.65 for chitosan containing *PI*CDH and *Ps*CDH). This confirms the presence of proteins in these samples.

#### 2.2.2. Examination of the Sample Surface Using the SEM Microscopy Technique

**Figure 3:**
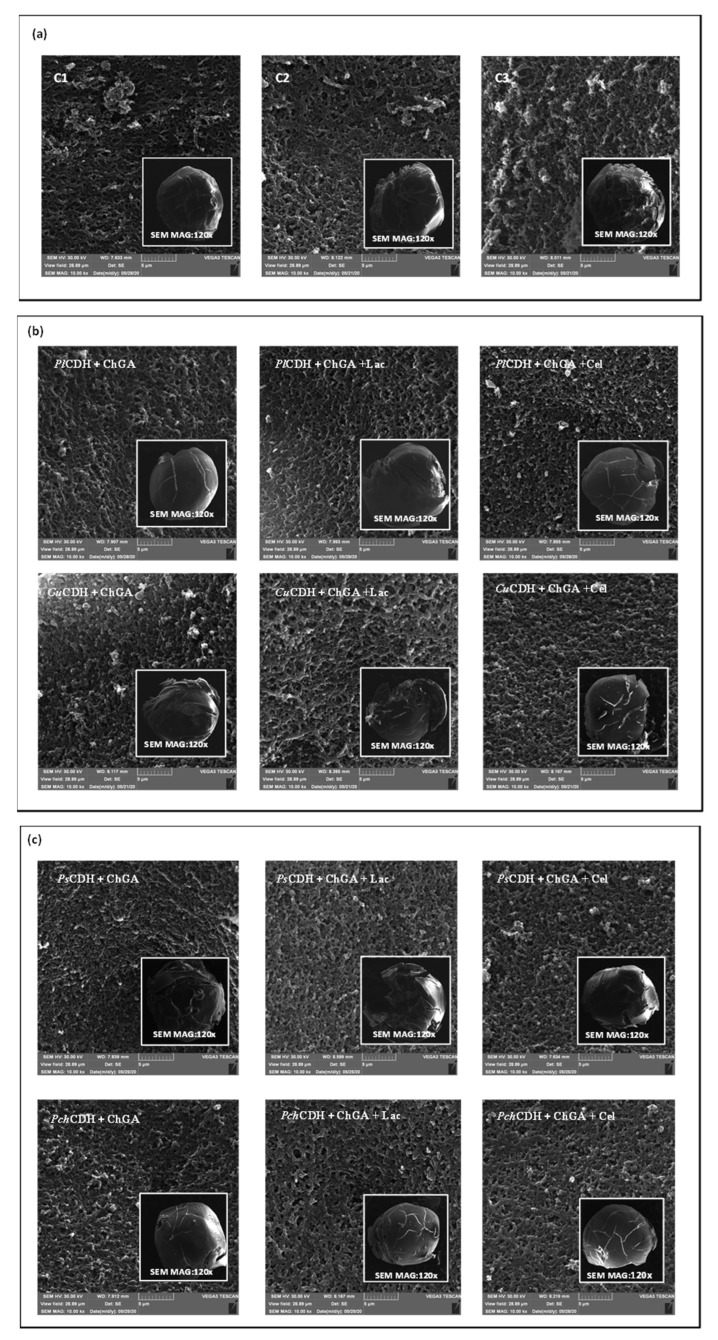
(**a**) SEM micrographs of the surface of chitosan with glutaraldehyde (ChGA) (C1-control), ChGA + Lac (C2-control), ChGA + Cel, (C3-control); (**b**) SEM micrographs of the surface of chitosan with glutaraldehyde (ChGA) containing CDHs isolated from *P. lindtneri*: *Pl*CDH + ChGA (1a), *Pl*CDH + ChGA + Lac (1b), *Pl*CDH + ChGA + Cel (1c) and from *C. unicolor*: *Cu*CDH + ChGA (2a), *Cu*CDH+ ChGA + Lac (2b), *Cu*CDH + ChGA + Cel (2c); (**c**) SEM of the surface of chitosan with glutaraldehyde (ChGA) (containing CDHs isolated from *P. sanguineus*: *Ps*CDH + ChGA (3a), *Ps*CDH + ChGA + Lac (3b), *Ps*CDH + ChGA +Cel (3c) and from *P. chrysosporium*: *Pch*CDH + ChGA (4a), *Pch*CDH+ ChGA + Lac (4b), and *Pch*CDH + ChGA + Cel (4c).

The study of the surface of many samples used in different biological systems (e.g., materials for the immobilization of cells or enzymes) requires the use of modern and precise tools, including microscopic techniques such as SEM [51]. The microscopic analysis of the surface of chitosan beads modified with glutaraldehyde, CDH (*Pl*CDH + ChGA, *Cu*CDH+ ChGA, *Ps*CDH + ChGA, *Pch*CDH + ChGA), lactose, and cellobiose evidenced morphological changes in all of the analyzed samples (Figure 3). Chitosan beads with glutaraldehyde (ChGA) and additionally bound with lactose (ChGA + Lac) and cellobiose (ChGA + Cel) were used as controls. During the experiments, it was observed that even in the case of the control without the CDH enzyme, the introduction of lactose or cellobiose significantly weakened the integrity of the surface of the tested biomaterials. Most likely as a result of the immobilization procedure, the beads exhibited clear cracks and a loss of shape (Figure 3a). Interestingly, the SEM detection showed that the introduction of *P. lindtneri* and *P. chrysosporium* CDH into the composition of the chitosan beads resulted in the smoothing of the biomaterial surface in comparison to the control, which suggests an improvement in the mechanical properties of the obtained samples (Figure 3b,c). It should be underlined that, in the case of *P. chrysosporium*, the presence of cellobiose additionally strengthened the surface of the chitosan beads, whereas no such effect was noted in the case of *P. lindtneri*. Contrarily, in the case of the *C. unicolor* and *P. sanguineus* enzymes, the modification of the composition of the chitosan beads by the immobilization of CDH and the addition of lactose and cellobiose caused negative changes in the mechanical properties of the tested biomaterials. The structure and shape of the surface of the modified beads were clearly disturbed, which may also suggest a reduction in their resistance to stress conditions present during their catalytic activity. In both cases, the presence of bound lactose further intensified this effect, and the bound cellobiose molecules seemed to weaken the effect by improving the integrity of the outer layer of the modified carrier (Figure 3b,c).

Other papers examining the surface morphology of normal and activated chitosan beads as enzyme carriers found increased roughness after treatment with glutaraldehyde and enzyme immobilization [52,53]. Another study suggested that the porosity of the surface increased after the use of glutaraldehyde [54].

#### 2.2.3. Antioxidant Properties

**Figure 4:**
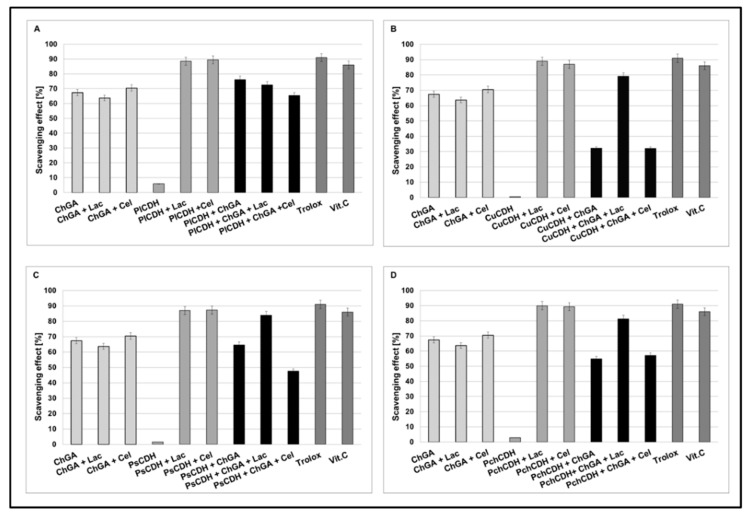
Scavenging effects of chitosan beads with glutaraldehyde (ChGA) (C1-control) with lactose ChGA + Lac (C2-control), or cellobiose ChGA + Cel (C3-control) containing CDHs isolated from *Phlebia lindtneri (Pl*CDH) (**A**), *Cerrena unicolor (Cu*CDH) (**B**), *Pycnoporus sanguineus* (*Ps*CDH) (**C**) and *Phanerochaete chrysosporium (Pch*CDH) (**D**). Data are mean ± SD for three measurements (n = 3).

Fungi are known for the production of substances with potent antioxidant properties such as vitamins, carotenoids, polyphenols, polysaccharides, minerals, and enzymes. In our earlier studies, fungal cellobiose dehydrogenases have been reported to exhibit strong antioxidant activity in the presence of lactose and cellobiose substrates [55,56]. Although chitosan is known rather for its antibacterial properties, its antioxidant properties have been confirmed in some studies. Yen Ming-Tsung et al. observed antioxidant activities at a level of 58.3–70.2% for 1 mg/m chitosan from crab shells [57]. Additionally, the antioxidant activity of chitosan can be enhanced by synthesizing chitosan derivatives. For example, Chatterjee et al. showed that native chitosan with MW = 100 kDa exhibited no inhibition against DPPH in contrast to four new phenolic conjugates with significantly improved antioxidant activities (50 % inhibition of 2,2′-diphenyl-1-picrylhydrazyl radical and hydroxyl free radical) [58]. Simultaneously, the commercial chitosan carrier used in the immobilization process also showed antioxidant properties, which is confirmed by the results of our study presented in Figure 4.

In order to compare the antioxidant activities of CDHs immobilized on the chitosan carrier with the activities of native CDHs, the DPPH-scavenging method with Trolox and vitamin C as a control model system was used. Our result showed that, in the presence of lactose or cellobiose as substrate, all the native CDHs had very good antioxidant properties (about 80 to 90% of scavenging). As a result of the CDH immobilization on chitosan carriers, the appearance of antioxidant properties was also observed in the case of the enzyme without the presence of the substrate (about 30 to 78% of scavenging). This is probably related to the antioxidant properties of chitosan. In turn, a decrease in the antioxidant properties (against the control) was observed for the preparations with CDH immobilized on the chitosan carrier, especially in the presence of the cellobiose substrate (about 30 to 60 % of scavenging). The least significant decrease in the antioxidant activity after the immobilization process was observed for CDHs isolated from *Phlebia lindtneri (Pl*CDH) (Figure 4A). The decrease in the antioxidant activity of the studied enzymes against the control samples can be explained by the steric effect and structural changes in the protein conformation induced by the immobilization process [42]. These changes may have significant effects on the enzymatic activity of CDH. It should be emphasized that the structure of the enzyme molecule is the key of catalytic importance in this case for the production of lactobionic or cellobionic acid and hydrogen peroxide as a byproduct. In our previous studies, we demonstrated that CDHs without substrates (lactose and cellobiose) did not exhibit antioxidant properties. Reaction products were formed only in the presence of enzyme reaction substrates. These products are probably responsible for the antioxidant properties [28]. Thus, the antioxidant properties of CDH appear during the reaction with specific substrates. As a result of the immobilization process, the structure of the protein is probably changed or the active chemical groups responsible for the catalytic properties of the enzyme are partly blocked, which may explain the decrease in the antioxidant activity of the studied CDHs in comparison to the control samples [42].

#### 2.2.4. Operational Stability of Chitosan Beads

**Figure 5:**
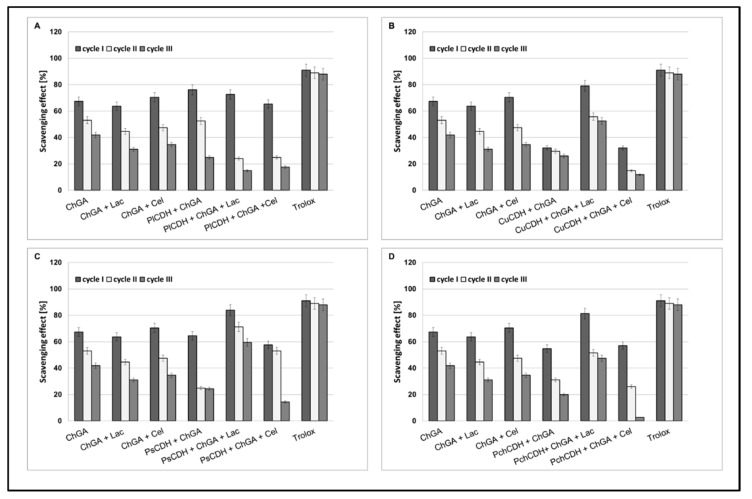
Operational stability of chitosan beads with glutaraldehyde (ChGA) (C1-control) with lactose, ChGA + Lac (C2-control), or cellobiose ChGA + Cel, (C3-control) containing CDHs isolated from *Phlebia lindtneri (Pl*CDH) (**A**), *Cerrena unicolor (Cu*CDH) (**B**), *Pycnoporus sanguineus* (*Ps*CDH) (**C**) and *Phanerochaete chrysosporium (Pch*CDH) (**D**). Data are mean ± SD for three measurements (n = 3).

Operational stability is an important parameter determining the usefulness of immobilization techniques in industry. The enzyme half-life (t_½_) is a parameter characterizing the operational stability of preparations after immobilization. It is a quantity describing a period after which half of the initial catalytic activity of the enzyme is lost. In our study, we tested the antioxidant operational stability of CDH immobilized on chitosan beads. In all the tested samples, the best results were obtained for preparations containing lactose as a substrate, except chitosan beads containing CDH isolated from *Phlebia lindtneri (Pl*CDH). The most promising results in the three cycles of determination (about 60% and 58% of scavenging) were obtained for *Ps*CDH + ChGA + Lac and *Cu*CDH+ ChGA + Lac (Figure 5).

Our previous research on the immobilization of CDHs on alginate beads showed approx. only a 35% and 20% scavenging effect of CDH from *Phanerochaete chrysosporium* and *Phlebia lindtneri* [28]. In turn, Yang et al. immobilized cellobiose dehydrogenase from *Aspergillus fumigatus* (*Af*CDH) and laccase separately on magnetic chitosan spheres. In this multienzymatic system, they achieved 70 % relative catalytic efficiency after 10 cycles of reuse [7]. The loss of enzymatic activity after the immobilization process may depend on many factors, such as the elution of the biocatalyst from the support, blocking of the matrix pores, and destruction of the support material [59]. Good operational stability of enzymes is a very important parameter, as it facilitates multiple uses of the bound enzyme in the production process, which significantly reduces production costs. The ability of CDHs immobilized on chitosan beads to maintain enzymatic activity in several cycles of reuse is an advantage over the native form of this enzyme.

#### 2.2.5. In Vitro Cytotoxicity Assessment

**Figure 6:**
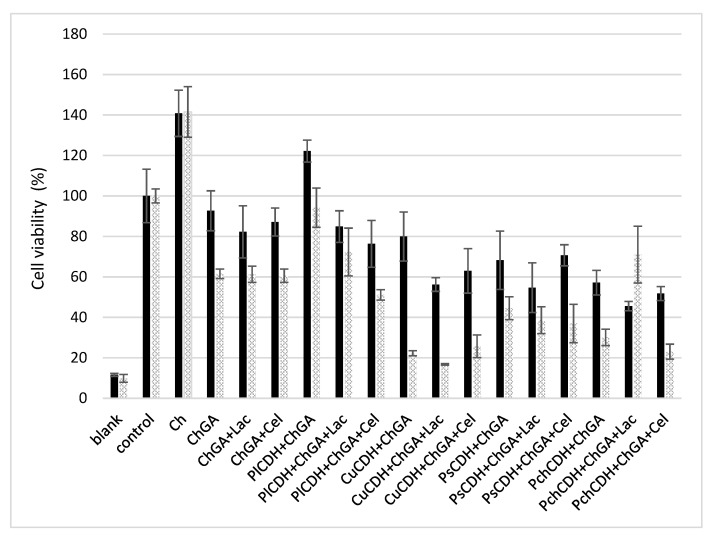
MTT cell viability assay of Vero cells incubated for 24 and 48 h with different chitosan beads: without glutaraldehyde (Ch), with glutaraldehyde (ChGA), with lactose (ChGA + Lac), or cellobiose (ChGA + Cel), containing CDHs isolated from *Phlebia lindtneri* (*Pl*CDH), *Cerrena unicolor* (*Cu*CDH), *Pycnoporus sanguineus* (*Ps*CDH), and *Phanerochaete chrysosporium* (*Pch*CDH)**.** Data are mean ± SD for three measurements (n = 3).

The cytotoxic effect of chitosan beads with immobilized CDH and the CDH/substrate system was investigated using healthy mammalian Vero cells. To assess the toxicity of the beads with the different immobilized CDH systems (with or without the substrate) presented in this work, MTT viability cell assays were conducted in 24 h and 48 h incubation periods. The results obtained are shown in Figure 6. At first, the toxicity of chitosan beads without the addition of the enzyme activated and not activated with glutaraldehyde also in the presence of the substrates (lactose and cellobiose) was verified. Chitosan beads that were not activated with glutaraldehyde (Ch) exerted a strong proliferative effect on the cell line studied. On the other hand, the glutaraldehyde-activated beads showed low cytotoxicity after the 24 h incubation (less than 20% growth inhibition). After the 48 h incubation, cell viability decreased by another 20%. Similar levels of cell viability were obtained in the variant with the enzyme from *P. lindtnerii* in the presence of the substrates (PlCDH + ChGA + Lac and PlCDH + ChGA + Cel), while PlCDH without the substrate (PlCDH + ChGA) caused the proliferation of the cells. In the case of the other enzymes, there was a decrease (20–40%) in cell viability after 24 h of incubation. In addition, it was found that the enzyme from *C. unicolor* (CuCDH) had the most toxic effect on Vero cells after 48 h both in the presence and absence of the substrates. No research on the cytotoxicity of CDHs or products of enzymatic reactions (aldobionic acid and H_2_O_2_) has been performed so far. Our research team has done a series of such analyses, and they will be published shortly. However, research on the cytotoxicity of *N*, *N*, *N*-trimethyl chitosan/alginate beads containing gold nanoparticles was carried out by Martins et al. (2015). They showed that this material was slightly destructive for healthy VERO cells, with viability reduced to 75%. On the other hand, raw beads exhibited quite good biocompatibility with the healthy Vero line, as approximately 100% of the total cells remained viable after 24 h of incubation [60].

The antimicrobial effects of CDHs immobilized on chitosan beads and the CDH/substrate systems from the same four fungi were tested against two strains of Gram-negative bacteria and two strains of Gram-positive bacteria. The assessment of the antibacterial activity of the tested CDHs and the CDH/substrate systems is presented in Table S1. The minimum inhibitory concentrations (MIC) of free CDHs (with and without the substrate) used for the immobilization process is presented in Table S2.

## 3. Materials and Methods

### 3.1. Microorganisms

Fungal strains producing cellobiose dehydrogenase (CDH) were taken from the collection at the Department of Biochemistry of Maria Curie, Sklodowska University (Poland) as described in previous publications [28].

### 3.2. Materials

The chitosan (medium molecular weight, degree of deacetylation 75–85%) and the glutaraldehyde (25% (*v*/*v*)) aqueous solution were obtained from Sigma Aldrich (Steinheim, Germany). Media components and other chemicals were purchased from Merck (Darmstadt, Germany), VWR (Vienna, Austria), BioRad (Warsaw, Poland), POCH (Gliwice, Polska), or BioMaxima (Lublin, Poland). All reagents and chemicals used in the work were of the highest available purity and analytical grade. Deionized water was used for the preparation of all aqueous solutions.

### 3.3. Enzyme Preparation

The preparation of semi-pure cellobiose dehydrogenase was carried out using a two-step purification strategy according to Sulej et al. (2019) [51]. Briefly, the crude enzyme was precipitated with ammonium sulfate fractionation in the saturation ranges of 40–90% (*P. sanguineus*), 30–50% (*P. lindtneri*), 15–85% (*C. unicolor*), and 20–80% (*P. chrysosporium*) at 0 °C. The precipitates were dissolved in deionized water and by diafiltration through centrifugal concentrators (Vivaspin Turbo 15) in polyethersulphone (PES) with a cut-off of 30 kDa (Sartorius, Göttingen, Germany). The diafiltrated sample was loaded onto a DEAE Sepharose (fast flow) column for anion exchange chromatography. Fractions with high CDH activity were pooled, desalted, and used as a partially purified enzyme for the immobilization experiments. The protein concentration during enzyme purification was monitored by ultraviolet (UV) absorbance at 280 nm.

### 3.4. Enzyme Activity Assay and Protein Determination

Cellobiose dehydrogenase activity was determined by the lactose-dependent reduction of 2,6-dichloroindophenol (DCIP) (Sigma Chemical Co., St. Louis, MO, USA) at 520 nm (ε_520_ = 6.8 mM^−1^·cm^−1^), pH 4.5, and 30 °C. The method developed by Baminger et al. (1999) [61] with modifications described in our previous work was used [28]. The protein concentration was determined using the Bradford method [62] with crystalline bovine serum albumin (BSA) as a standard.

### 3.5. Immobilization of Cellobiose Dehydrogenase on Chitosan Beads

#### 3.5.1. Preparation of Chitosan Beads

Chitosan powder (3 g) was dissolved in 100 mL of an acetic acid solution (2%, *v*/*v*) and mixed on a magnetic stirrer for 1 h at room temperature. Beads were formed by extruding the solution through a syringe needle into a coagulant bath of 1 M NaOH solution with constant stirring. The beads were kept in a 1 M NaOH solution for 24 h. After hardening, the pretreated chitosan beads were collected by filtration, washed with deionized water until neutrality, and stored at 4 °C.

#### 3.5.2. Direct Adsorption

Chitosan beads (0.5 g) were added into 5 mL of a CDH solution (1 mg/mL). CDHs for immobilization were prepared by mixing with water and lactose or cellulose in a 1:1 ratio. The solution was stirred with chitosan beads at 25 °C, 100 rpm for 3.0 h and kept in the fridge overnight. In the next step, the chitosan beads with the adsorbed enzyme were separated and washed three times with 100 mM phosphate-citric acid buffer (pH 5.0) to remove unbound enzymes.

#### 3.5.3. Covalent Immobilization Using Glutaraldehyde/Activation of Chitosan Beads

CDHs were immobilized on chitosan beads using glutaraldehyde as a linker. The chitosan beads (support) were activated by treatment thereof with 5% concentrations of glutaraldehyde and cross-linking (25 °C, 100 rpm, 1 h). After activation, the beads were washed thoroughly with distilled water to remove glutaraldehyde. The enzymes for immobilization were prepared in the same way as before. The immobilization process was carried out for 3 h at room temperature in a shaking incubator (100 rpm) and then the preparations were left overnight at 4 °C. The next day, the beads were washed three times with 100 mM phosphate-citric acid buffer (pH 5.0) to remove any unbound enzyme.

The percentage of immobilization was calculated as follows:

Total activity obtained in beads after immobilization/Total activity of the soluble enzyme loaded) × 100.

**Figure 7:**
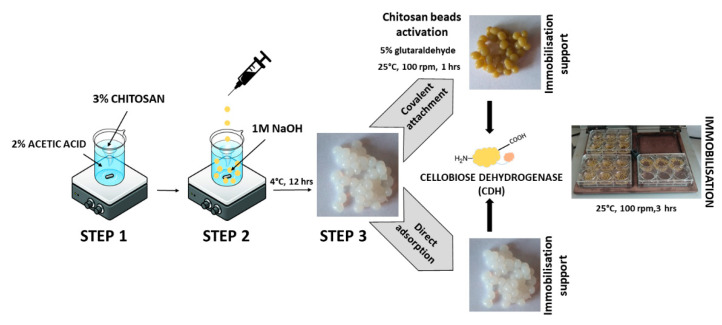
Steps in the immobilization of cellobiose dehydrogenase on chitosan beads.

The scheme of preparation, activation, and immobilization of CDH on chitosan beads is shown in Figure 7.

### 3.6. Characterization of Chitosan Beads

#### 3.6.1. FTIR

The FTIR-ATR analysis of the samples was performed using a Vertex 70 spectroscope (Bruker, Billerica, MA, USA) equipped with an ATR-diamond crystal within the range of 4000–370 cm with a 1 cm resolution. In total, 32 scans were collected for each sample. Prior to the measurement, all samples were dried in an exicator for 24 h to remove the excess unbound water. Spectra were analyzed using OPUS 7.0 program (Bruker, Billerica, MA, USA). The ratio of amide I to amide II bands was calculated in the same program using the values of 1650 cm^−1^ and 1564 cm^−1^ band intensity relative to the local baseline.

#### 3.6.2. Scanning Microscopy (SEM) of Modified Chitosan Beads

The surface of the freeze-dried chitosan beads modified by immobilization of CDH isolated from the four fungal strains, lactose, and cellobiose was visualized using the scanning microscopy technique (SEM) at 30 kV accelerating voltage. Beads containing chitosan with glutaraldehyde (ChGA), ChGA with immobilized lactose, and ChGA with bounded cellobiose were the controls. All of the samples were coated with gold (Sigma-Aldrich, St. Louis, MO, USA) and their morphologies were observed using VEGA-3 LMU SEM (TESCAN, Brno, Czech Republic). The magnification ×10,000 was used to examine the surface of the control and the CDH-, laccase-, and cellobiose-modified samples. In order to check and confirm possible changes in the image of the samples, 10 separate areas with chitosan beads were selected.

#### 3.6.3. Antioxidant Properties of CDH Immobilized on Chitosan Beads

The DPPH free radical scavenging activity of free and chitosan-bound cellobiose dehydrogenases (CDH) was determined with the method described by Paduch et al. (2008) [63] with some modification. The analyzed samples (0.1 mL or 0.1 mg chitosan beads, 6.25 to 800 µg/mL) were added to 0.1 mL of a DPPH^.^ solution (0.2 mg/mL in ethanol). Trolox and vitamin C, the well-known standards with strong antioxidant activities, were used as positive controls. Absorbance at 515 nm was determined at room temperature after 15 min (optimal time of incubation). The percentage of inhibition of DPPH oxidation was calculated as follows:

*DPPH scavenging effect* (%) = [(*A*_0_ − *A*_1_)/*A*_0_] × 100

where *A*_0_ means the absorbance of the control sample and *A*_1_ means the absorbance of the standard or the tested compound. The standard (Trolox and Vitamin C) calibration curves were prepared for a concentration range from 6.25 to 800 µg/mL.

#### 3.6.4. Operational Stability

The antioxidant operational stability of the immobilized CDH was assessed by incubating chitosan beads (~0.1 mg) in 0.1 mL of a DPPH^.^ solution (0.2 mg/mL in ethanol). After 15 min of incubation, the absorbance of the DPPH^.^ solution was measured. The chitosan beads were washed five times with 0.1 M phosphate buffer pH 6.5 and suspended in a new solution of DPPH to begin the next cycle of activity measurement.

#### 3.6.5. Cytotoxicity Evaluation

Cell viability was analyzed with the 3-(4,5-dimethylthiazol-2-yl)-2,5-diphenyltetrazolium bromide (MTT) method. The cytotoxicity of beads with immobilized CDHs and CDH/substrate systems was investigated as in Huber et al. (2017) [64] with several modifications. The beads were UV-sterilized for 1 h in sterile conditions. The sterile beads were then incubated for 24 h in DMEM medium at 37 °C and 5% CO_2_ to allow leaching into the medium. Afterward, the medium was filtered through a 0.22-μm filter and the leach-out medium was used for cytotoxicity testing. The extracts were added into the wells of a 96-well plate containing 24 h Vero (African Green Monkey-kidney, ATCC, Kennett Square, PA, USA) cell line solutions (~1 × 10^4^ cell/well) and incubated for 24 and 48 h. A control was performed by using untreated medium. After incubation, 10 µL of 5 mg/mL MTT (Sigma-Aldrich) was added to each well for 3 h incubation in darkness at room temperature, after which the medium was removed and formazan crystals within the cells were solubilized by 100 µL/well of DMSO (Chempur). The plates were shaken to fully dissolve the crystals. The optical density at 570 nm was detected with a Thermo Scientific Multiscan FC spectrophotometer. The results are presented as a percentage of the control values from three separate wells.

#### 3.6.6. Statistical Analysis

The presented results are expressed as mean ± SD from three independent experiments (n = 3). The mean values and standard deviation were calculated using one-way ANOVA (Statgraphics Online); next, the means were compared using Tukey’s multiple range test. The Excel program (Microsoft Office 365 package) was used for the calculation of the data. Values of *p* ≤ 0.05 were considered statistically significant.

## 4. Conclusions

This paper presents a proposal for an innovative and efficient system for exploiting the catalytic potential of cellobiose dehydrogenase based on the immobilization of the enzyme on chitosan as an immobilization matrix. For the most complete demonstration of the possibilities of the proposed experimental setup, two different methods (adsorption and covalent binding) of immobilization of CDH on chitosan beads were tested. Although the adsorption method seems to be less invasive, the chemical bonding method using glutaraldehyde is definitely more efficient. As indicated by the obtained results, it is possible to obtain almost 90% binding efficiency while maintaining the most important catalytic properties. It should be emphasized that the physicochemical properties of the chitosan beads were confirmed by multi-plane analyses, including the FTIR and SEM techniques. It was shown that the immobilized enzyme in the proposed form of modification had good operational stability, which justifies its economical use in industry and biotechnology. It is particularly worth emphasizing that immobilized CDH maintains antioxidant, antibacterial, and cytotoxic properties at a level comparable to its free form. Based on the data obtained in this study, it can definitely be confirmed that chitosan is a promising and valuable material for the development of innovative immobilized CDH systems for biomedical applications or food packaging.

## Figures and Tables

**Table 1:** Efficiency of cellobiose dehydrogenase adsorption on the chitosan support (Ch) containing CDHs isolated from *P. lindtneri*: *Pl*CDH + Ch, with lactose *Pl*CDH + Ch + Lac, with cellobiose *Pl*CDH + Ch + Cel, from *C. unicolor*: *Cu*CDH + Ch, *Cu*CDH + Ch + Lac, *Cu*CDH + Ch + Cel, from *P. sanguineus*: *Ps*CDH + Ch, *Ps*CDH + Ch+ Lac, *Ps*CDH + Ch + Cel, and from *P. chrysosporium*: *Pch*CDH + Ch, *Pch*CDH + Ch + Lac, *Pch*CDH + Ch + Cel.

Sample Type	Applied Protein [mg/g Carrier]	Bound Protein [mg/g Carrier]	Protein Yield [%]	CDH Activity before Immobilization [U/g Carrier]	Activity of CDH Bound with Chitosan [U/g Carrier]	Activity Yield [%]
PlCDH + Ch	8.25 ± 0.02	1.76 ± 0.02	21.35	14.21 ± 0.03	0	0
PlCDH + Ch + Lac	8.23± 0.02	2.24 ± 0.02	27.21	15.27 ± 0.03	7.07 ± 0.04	46.29
PlCDH + Ch + Cel	9.61 ± 0.02	3.18 ± 0.01	33.14	15.28 ± 0.03	2.24 ± 0.02	14.64
CuCDH + Ch	6.49 ± 0.02	2.68 ± 0.02	41.27	37.53 ± 0.08	0.39 ± 0.001	1.04
CuCDH + Ch + Lac	5.76 ± 0.01	3.12 ± 0.03	54.12	40.17 ± 0.06	31.62 ± 0.08	78.74
CuCDH + Ch + Cel	6.18 ± 0.02	2.97 ± 0.03	48.04	36.93 ± 0.05	16.04 ± 0.05	43.44
PsCDH + Ch	8.19 ± 0.02	2.12 ± 0.02	25.91	17.10 ± 0.03	0	0
PsCDH + Ch + Lac	9.23 ± 0.03	4.77 ± 0.03	51.72	17.88 ± 0.03	6.60 ± 0.03	36.93
PsCDH + Ch + Cel	8.90 ± 0.02	6.12 ± 0.03	68.73	16.65 ± 0.03	2.54 ± 0.02	15.28
PchCDH + Ch	9.70 ± 0.03	5.89 ± 0.04	60.68	12.58 ± 0.02	1.56 ± 0.02	12.37
PchCDH + Ch + Lac	10.17 ± 0.04	2.59 ± 0.02	25.44	11.27 ± 0.03	2.11 ± 0.01	18.74
PchCDH + Ch + Cel	10.41 ± 0.04	3.27 ± 003	31.45	14.55 ± 0.03	2.66 ± 0.02	18.30

**Table 2:** Efficiency of cellobiose dehydrogenase immobilization with glutaraldehyde on the chitosan support (ChGA) containing CDHs isolated from *P. lindtneri*: *Pl*CDH + ChGA, with lactose *Pl*CDH + ChGA + Lac, with cellobiose *Pl*CDH + ChGA + Cel, from *C. unicolor*: *Cu*CDH + ChGA, *Cu*CDH + ChGA + Lac, *Cu*CDH + ChGA + Cel, from *P. sanguineus*: *Ps*CDH + ChGA, *Ps*CDH + ChGA + Lac, *Ps*CDH + ChGA + Cel, and from *P. chrysosporium*: *Pch*CDH + ChGA, *Pch*CDH + ChGA + Lac, *Pch*CDH + ChGA + Cel).

Sample Type	Applied Protein [mg/g Carrier]	Bound Protein [mg/g Carrier]	Protein Yield [%]	CDH Activity before Immobilization [U/g Carrier]	Activity of CDH Bound with Chitosan [U/g Carrier]	Activity Yield [%]
PlCDH + ChGA	8.25 ± 0.02	3.58 ± 0.02	43.37	14.21 ± 0.03	4.41 ± 0.02	31.03
PlCDH + ChGA + Lac	8.23 ± 0.02	5.05 ± 0.03	61.36	15.27 ± 0.03	15.14 ± 0.06	99.18
PlCDH + ChGA + Cel	9.61 ± 0.02	6.85 ± 0.02	71.32	15.28 ± 0.03	13.64 ± 0.05	89.23
CuCDH + ChGA	6.49 ± 0.02	6.04 ± 0.04	93.06	37.53 ± 0.08	34.63 ± 0.08	92.28
CuCDH + ChGA + Lac	5.76 ± 0.01	3.57 ± 0.02	61.87	40.17 ± 0.06	29.60 ± 0.05	73.69
CuCDH + ChGA + Cel	6.18 ± 0.02	4.67 ± 0.01	75.49	36.93 ± 0.05	28.55 ± 0.04	77.31
PsCDH + ChGA	8.19 ± 0.02	3.95 ± 0.03	48.29	17.10 ± 0.03	4.89 ± 0.02	28.59
PsCDH + ChGA + Lac	9.23 ± 0.03	7.65 ± 0.05	82.87	17.88 ± 0.03	16.45 ± 0.03	92.02
PsCDH + ChGA + Cel	8.90 ± 0.02	7.07 ± 0.04	79.39	16.65 ± 0.03	12.91 ± 0.02	77.55
PchCDH + ChGA	9.70 ± 0.03	3.57 ± 0.02	36.76	12.58 ± 0.02	7.53 ± 0.03	59.84
PchCDH + ChGA + Lac	10.17 ± 0.04	7.76 ± 0.03	76.34	11.27 ± 0.03	10.13 ± 0.04	89.85
PchCDH + ChGA + Cel	10.41± 0.04	9.29 ± 0.03	89.28	14.55 ± 0.03	12.24 ± 0.04	84.16

## Data Availability

Not applicable.

## Supplementary Materials

The supporting information can be downloaded at: https://www.mdpi.com/article/10.3390/ijms24054535/s1. References [28,33,65,66,67,68,69,70,71] are cited in supplementary materials.
