# Supplementary material for: Chitosan as a Promising Support of a CDH Activity Preservation System for Biomedical and Industrial Applications
Source: Int J Mol Sci. 2023 Feb 25;24(5):4535. doi: 10.3390/ijms24054535 (PMC10003442; doi:10.3390/ijms24054535)
Supplement: Supplementary file 1 [file ijms-24-04535-s001.zip › ijms-2218737-supplementary.pdf]

Supplementary material

Antimicrobial properties

Enzymes are now increasingly being used in antimicrobial systems in view of their ability to directly attack microorganisms, interfere with biofilm formation, destroy biofilm, and/or catalyze reactions that lead to the production of antimicrobial compounds [65]. These enzymes represent a new generation of antimicrobial agents. Glucose oxidase is the most well-known antimicrobial enzyme. It is increasingly being investigated in terms of its application in the food industry to kill food spoiling microorganisms and food borne pathogens. However, in recent years, cellobiose dehydrogenase has been gaining increasing biotechnological importance as an antimicrobial and antioxidant agent [33,66]. As in the case of glucose oxidase, the antimicrobial properties of the cellobiose dehydrogenase/substrate (lactose or cellobiose) system are based on the release of H_2O_2 and accumulation of corresponding aldobionic (lactobionic or cellobionic) acid. Hydrogen peroxide kills bacterial cells through peroxidation and disruption of cell membranes, oxidation of oxygen scavengers and thiol groups, and disruption of protein synthesis [67]. Aldobionic acid lowers the pH in the environment, which has a negative effect on bacterial growth. Recently, our group has demonstrated the ability of free cellobiose dehydrogenase (CDH) isolated from *P. lindtneri*, *C. unicolor*, *P. sanguineus*, and *P. chrysosporium* and CDH/substrate systems to inhibit bacterial growth [28]. In the present study, the antimicrobial effects of CDHs immobilized on chitosan beads and the CDH/substrate systems from the same four fungi were tested against two strains of Gram-negative bacteria (*E. coli* ATCC 25922, *P. aeruginosa* ATCC 27853) and two strains of Gram-positive bacteria (*S. aureus* ATCC 25923, *S. epidermidis* ATCC 14990). The assessment of the antibacterial activity of the tested CDHs and the CDH/ substrate systems is presented in Table S1. The minimum inhibitory concentrations (MIC) of free CDHs (with and without the substrate) used for the immobilization process is presented in Table S2.

Table S1 Antimicrobial activity of cellobiose dehydrogenase immobilized on a chitosan support activated with glutaraldehyde (ChGA). CDHs were isolated from *P. lindtneri*: PlCDH + ChGA, with lactose PlCDH + ChGA + Lac, with cellobiose PlCDH + ChGA + Cel, from *C. unicolor*: CuCDH + ChGA, CuCDH + ChGA + Lac, CuCDH + ChGA + Cel, from *P. sanguineus*: PsCDH + ChGA, PsCDH + ChGA + Lac, PsCDH + ChGA + Cel, and from *P. chrysosporium*: PchCDH +.ChGA, PchCDH + ChGA + Lac, PchCDH + ChGA + Cel)

**Sample type**	**Percentage of growth inhibition [%]**			
	E. coli (ATCC 25922)	*S. aureus* (ATCC 25923)	*P. aeruginosa* (ATCC 27853)	*S. epidermidis* (ATCC 14990)
**PlCDH + Ch**	0±0.01	40±0.02	25±0.04	79±0.04
**PlCDH + Ch + Lac**	11±0.02	4±0.03	34±0.06	100±0.01
**PlCDH + Ch + Cel**	24±0.03	25±0.03	17±0.02	92±0.02
**CuCDH + Ch**	20±0.06	38±0.06	0±0.01	65±0.02
**CuCDH + Ch + Lac**	43±0.02	94±0.13	93±0.11	49±0.05
**CuCDH + Ch + Cel**	12±0.01	27±0.08	52±0.02	27±0.01
**PsCDH + Ch**	16±0.05	48±0.12	47±0.01	100±0.02
**PsCDH + Ch + Lac**	23±0.02	97±0.09	52±0.03	100±0.04
**PsCDH + Ch + Cel**	23±0.03	17±0.03	38±0.08	100±0.05
**PchCDH + Ch**	16±0.06	46±0.08	12±0.02	100±0.02
**PchCDH + Ch + Lac**	24±0.01	45±0.09	30±0.04	100±0.03
**PchCDH + Ch + Cel**	19±0.04	41±0.03	35±0.06	100±0.06

Table S2 The Minimum Inhibitory Concentration (MIC) of cellobiose dehydrogenase CDH isolated from *P. lindtneri*: PlCDH with lactose PlCDH + Lac, with cellobiose PlCDH + Cel, from *C. unicolor*: CuCDH, CuCDH + Lac, CuCDH + Cel, from *P. sanguineus*: PsCDH, PsCDH + Lac, PsCDH + Cel, and from *P. chrysosporium*: PchCDH, PchCDH + Lac, PchCDH + Cel). Gentamycin is used as control.

**Sample type**	**MIC [mg/ml]**			
	E. coli (ATCC 25922)	*S. aureus* (ATCC 25923)	*P. aeruginosa* (ATCC 27853)	*S. epidermidis* (ATCC 14990)
**PlCDH**	5.00	3.00	4.00	1.00
**PlCDH + Lac**	5.00	2.00	3.00	1.00
**PlCDH + Cel**	5.00	3.00	3.00	1.00
**CuCDH**	3.00	5.00	5.00	2.00
**CuCDH + Lac**	2.00	0.25	0.25	0.15
**CuCDH + Cel**	5.00	2.00	2.00	0.25
**PsCDH**	5.00	5.00	5.00	2.00
**PsCDH + Lac**	2.00	1.00	1.00	0.25
**PsCDH + Cel**	3.00	5.00	1.00	0.25
**PchCDH**	5.00	5.00	5.00	1.00
**PchCDH + Lac**	2.00	1.00	2.00	0.25
**PchCDH + Cel**	5.00	5.00	5.00	0.50
**Gentamicin**	0.005	0.01	0.01	0.002

The results revealed the lowest effectiveness of PlCDH immobilized on chitosan beads towards almost all the tested organisms with the exception of *S. epidermidis*, whose growth was completely inhibited (100%) in the presence of the PlCDH/lactose system. *S. epidermidis* showed the highest sensitivity to all the investigated enzyme systems. The lowest level of inhibition in the range of 0 to 20% was achieved in the case of *E. coli* except for enzyme CuCDH in the presence of lactose as a substrate (43%). The other enzymes were effective in suppressing the growth of the bacteria with variable potency. The highest inhibition of the growth of *S. aureus*, i.e. 94 % and 97 %, was induced by CuCDH and PsCDH with lactose as a substrate, respectively, while much lower inhibition (45 %) was caused by the enzyme with PchCDH in the same conditions. The percent of *S. aureus* survival in the experiments with cellobiose as a substrate was 27%, 17%, and 41% in the CuCDH, PsCDH, and PchCDH variants, respectively. Comparable efficacy was observed in the case of *P. aeruginosa* incubated with CuCDH (93% and 52%), PsCDH (52% and 38%), and PchCDH (30% and 35%) in the presence of lactose and cellobiose substrates, respectively. The results of the antimicrobial activity of the four immobilized CDHs with different

substrates may suggest that the *S. epidermidis* strain was the most sensitive to CDH, in contrast to *E. coli*. The antibacterial activity of free CDHs used for the immobilization process was evaluated by determining MICs in relation to the some bacterial strains. The MIC values of CDHs with and without the substrate were in the range from 0.15 mg/mL to 5 mg/mL. The intensity of antibacterial activity varied depending on the species of bacteria. The high MIC values (0.15 to 5 mg/mL) of free CDH result from the mechanism of the enzymatic reaction described in our previous work [28] and are comparable to the values obtained in assessment of the antimicrobial properties of fungi or plant extracts. Gentamicin, which is a control in the MIC method, has a well-known mechanism of action that makes the antibiotic concentration much lower, reaching values of the order of µg/mL. Biomedical applications of CDH in the context of antimicrobial and antibiofilm properties were studied by Nyanhongo et al. (2017) [68]. System with recombinantly produced CDH from *Myriococcum thermophilum* in the presence of cellobiose successfully inhibited many common urinary catheter colonizing microorganisms, including the multidrug resistant *S. aureus, S. epiderimidis, Proteus mirabilis, Stenotrophomonas maltophilia, Acinetobacter baumanii,* and *P. aeruginosa* [69]. The use of immobilized CDH embedded in chitosan particles and the implementation of this system in lysozyme-reactive materials releasing CDH and its reaction products in the case of wound infection have been proposed for the treatment of chronic wounds [33,70]. The results obtained in our research team are very promising also in the context of potential biomedical applications, although they require further research and optimization.

Materials and Methods

Microorganisms

Antimicrobial activity tests were performed with strains of Gram-positive bacteria *Staphylococcus aureus* (ATCC 25923) and *Staphylococcus epidermidis* (ATCC 18112) and strains of Gram-negative bacteria *Escherichia coli* (ATCC 25922) and *Pseudomonas aeruginosa* (ATCC 27853). Bacteria used in the experiments were acquired from the culture collection of the Department of Genetics and Microbiology of Maria Curie-Sklodowska University (Poland).

<u>Antimicrobial activity</u>

The antibacterial activity of all the chitosan beads (~0.1 mg per well) with CDHs and CDH/substrate systems was tested against four bacterial strains: *S. aureus* (ATCC 25923), *S. epidermidis* (ATCC 18112), *E. coli* (ATCC 25922), and *P. aeruginosa* (ATCC 27853). The analyses were done with the broth microdilution method described by Wei et al. [71] with modifications. Beads with immobilized CDHs were placed into a 96-well plate. Each well contained 200 µL of Muller-Hinton broth inoculated with 10^4 CFU/mL of the tested bacterial strains. Microbial growth was determined after 18 h incubation at

37°C, the chitosan beads were removed, and the absorbance was measured at 550 nm using a microplate reader (TECAN Infinite M200 PRO) to assess the cell growth. All analyses were carried out in triplicate. The results were given in a percentage of bacterial inhibition (IC%) according to the equation below:

$$\textit{bacterial inhibition percentage (IC\%)} = 100 - (OD550_{assay}/OD550_{positive\ control}) \times 100$$

where OD550 is the optical density of the sample measured at 550 nm.

Determination of minimum inhibitory concentrations (MICs) of free CDHs

MICs of the CDHs were determined using the broth microdilution method as described by Wei et al. [9] with modifications. Free CDHs prepared for immobilization in a protein concentration of 10 mg/mL were used to determine MIC. Subsequently, 2-fold serial dilutions of each enzyme were prepared with Muller-Hinton broth medium at a volume of 200 mL per well in 96-well flat-bottom microtiter plates. The final concentrations of each CDH ranged from 0.1 to 5 mg/mL and that of gentamicin used as a positive control was from 1 to 20 µg/mL. The microtiter plate wells were inoculated with 10 mL per well of bacterial cell suspension at a final concentration of 10^4 CFU/ml for all bacterial species. After incubation at 37°C for 24 h, the absorbance was measured at 550 nm using a microplate reader (TECAN Infinite M200 PRO) to assess the cell growth. The minimum inhibitory concentration (MIC) values were defined as the lowest concentration of the tested agent that inhibited growth (no visible growth) or produced at least 80% reduction in absorbance compared with that of the enzyme or gentamicin free control. All assays were done in triplicate.

References

65. Augustin, M.; Ali-Vehmas, T.; Atroshi, F. Assessment of enzymatic cleaning agents and disinfectants against bacterial biofilms. *J Pharm Pharm Sci*. **2004** *18*, *7(1)*, 55-64.
33. Tegl, G.; Thallinger, B.; Beer, B.; Sygmund, C.; Ludwig, R.; Rollett, A.; Nyanhongo, G.S.; Guebitz, G.M. Antimicrobial cellobiose dehydrogenase-chitosan particles. *ACS Applied Materials & Interfaces* **2016**, *8*, 967-973.
66. Ludwig, R.; Harreither, W.; Tasca, F.; Gorton, L. Cellobiose dehydrogenase: a versatile catalyst for electrochemical applications. *ChemPhysChem* **2010**, *11*, 2674-2697.
67. Alt, E.; Leipold, F.; Milatovic, D.; Lehmann, G.; Heinz, S.; Schömig, A. Hydrogen peroxide for prevention of bacterial growth on polymer biomaterials. *The Annals of thoracic surgery* **1999**, *68*, 2123-2128.
28. Sulej, J.; Osińska-Jaroszuk, M.; Jaszek, M.; Grąz, M.; Kutkowska, J.; Pawlik, A.; Chudzik, A.; Bancerz, R. Antimicrobial and antioxidative potential of free and immobilised cellobiose dehydrogenase isolated from wood degrading fungi. *Fungal biology* **2019**, *123*, 875-886.
68. Nyanhongo, G.S.; Thallinger, B.; Guebitz, G.M. Cellobiose dehydrogenase-based biomedical applications. *Process Biochemistry* **2017**, *59*, 37-45.
69. Thallinger, B.; Argirova, M.; Lesseva, M.; Ludwig, R.; Sygmund, C.; Schlick, A.; Nyanhongo, G.S.; Guebitz, G.M. Preventing microbial colonisation of catheters: antimicrobial and antibiofilm activities of cellobiose dehydrogenase. *International journal of antimicrobial agents* **2014**, *44*, 402-408.

70. Öhlknecht, C.; Tegl, G.; Beer, B.; Sygmund, C.; Ludwig, R.; Guebitz, G.M. Cellobiose dehydrogenase and chitosan-based lysozyme responsive materials for antimicrobial wound treatment. *Biotechnology and Bioengineering* **2017**, *114*, 416-422.
71. Wei, G. X., Campagna, A. N., Bobek, L. A. Effect of MUC7 peptides on the growth of bacteria and on *Streptococcus* mutans biofilm. *Journal of antimicrobial chemotherapy* **2006**, 57(6), 1100-1109
